# Clinical Characteristics According to Age and Duration of Symptoms to Be Considered for Rapid Diagnosis of Pediatric Intussusception

**DOI:** 10.3389/fped.2021.651297

**Published:** 2021-03-31

**Authors:** In Kyu Park, Min Jeng Cho

**Affiliations:** Department of Surgery, Ulsan University Hospital, University of Ulsan College of Medicine, Ulsan, South Korea

**Keywords:** child, clinical characteristics, intussusception, bloody stool, abdominal pain (MeSH)

## Abstract

**Purpose:** The purpose of this study was to evaluate whether clinical findings in children with ileocolic intussusception differ based on age and duration of symptoms and to assess the clinical characteristics of diagnosed and undiagnosed patients to determine which symptoms make diagnosis more difficult.

**Methods:** We reviewed 536 medical records of <15-year-old children diagnosed with ileocolic intussusception between 2008 and 2019. We divided the children into three categories according to age (<1 year, 1–2 years, and ≥2 years). The children were also divided into two groups based on whether symptoms lasted for more or <6 h. Diagnosed and undiagnosed children were assessed separately during for the initial evaluation.

**Results:** Following analysis of the three age groups, bloody stool, post-enema bloody stool, diarrhea, vomiting, poor oral intake, and lethargy were more frequent in children aged <1 year. In children aged ≥2 years, non-specific abdominal pain was more frequent and the undiagnosed rate was higher. Following analysis of the duration of symptoms, paroxysmal pain was significantly more frequent in the early group (<6 h), and bloody stool and fever were significantly more frequent in the late group (≥6 h). Nonspecific abdominal pain was more frequent and the door-to-diagnosis time was significantly longer in the undiagnosed group than in the diagnosed group.

**Conclusions:** Clinical findings of ileocolic intussusception vary depending on the age and duration of symptoms. Younger children with paroxysmal pain, vomiting, bloody stool, poor oral intake, or lethargy should be suspected of having intussusception. In older children, non-specific abdominal pain without bloody stool may be a symptom of intussusception. Glycerin enema is helpful in diagnosing intussusception in children with no typical symptoms.

## Introduction

Intussusception is the most common cause of acute intestinal obstruction in infants and young children and is a potentially life-threatening condition that is commonly seen in the emergency room (ER) (1, 2). The incidence of intussusception in the pediatric population was reported to be 30–60 cases per 100,000 child-years in North America, Europe, and Australasia (3), while a higher incidence was reported in Asian countries, including South Korea, Vietnam, China, and Singapore (4, 5). In a recent nationwide study in South Korea, the incidences of intussusception in children under 1 and 2 year(s) of age were reported to be 193.2 and 196.7 per 100,000, respectively (6).

Untreated intussusception may lead to bowel necrosis and perforation, and even death (3, 7, 8). Early detection and intervention can significantly reduce the rates of mortality and morbidity. Screening suspected cases of intussusception in the ER is important, but the diagnosis can be difficult in children because of a non-specific presentation and physical examination. Traditional teaching states that intussusception presents with a classic triad of symptoms: paroxysmal abdominal pain, bloody stool, and vomiting (9, 10). However, this classic triad appears only in 10–20% of intussusception cases (2). Many patients present with only irritability or other non-specific complaints. Vomiting, a common presenting symptom, is also a common symptom in other gastrointestinal diseases. The isolated neurological symptomatology, including lethargy, convulsions, and sudden altered mental state, can appear as the initial presentation (11, 12). A major challenge in the diagnosis of intussusception is early determination of which patients are at low risk of intussusception based on clinical signs.

The primary objective of this study was to evaluate whether clinical findings in children with ileocolic intussusception differ based on age and duration of symptoms. The secondary objective was to evaluate the clinical characteristics of diagnosed and undiagnosed patients to determine which symptoms make diagnosis more difficult.

## Materials and Methods

### Study Design and Participants

**Figure 1:**
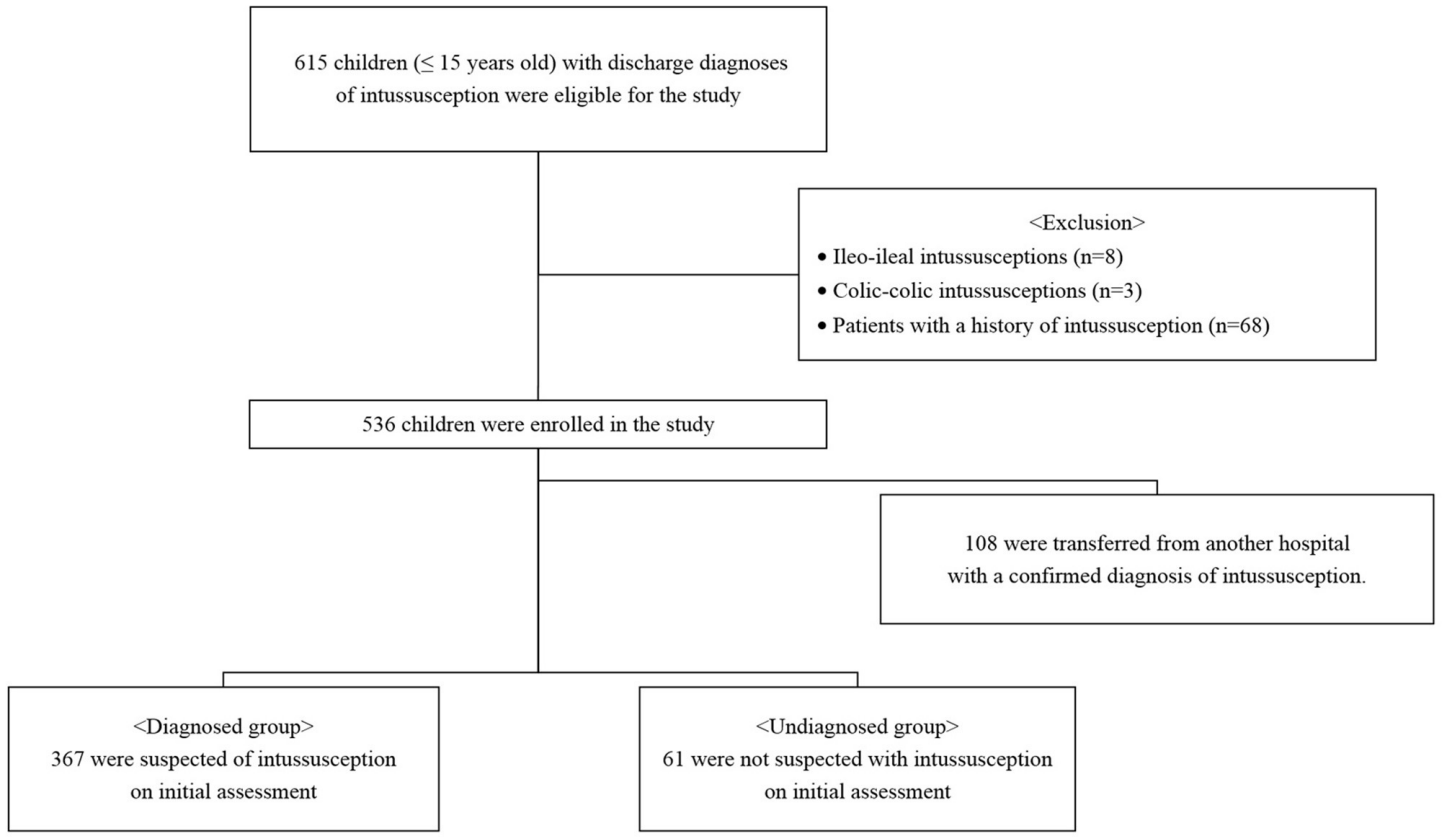
Flow chart of study population.

We retrospectively reviewed the medical records of children aged 0 to 15 years with discharge diagnoses of intussusception (10th revision of the International Statistical Classification of Diseases, K56.1) between January 2008 and December 2019 (Figure 1). All cases either presented directly to our ER or were transferred to the ER from referring peripheral hospitals for definitive care. Only patients with ileocolic intussusception were included in the study; patients with ileo-ileal and colic-colic intussusceptions were excluded. Patients with a history of intussusception were also excluded. This study was conducted in accordance with the principles embodied in the Declaration of Helsinki, and ethical approval was granted by our hospital's Human Research Ethics Committee (2020-09-008).

### Data Collection and Variable Definition

The following factors were extracted: demographic details and clinical features (abdominal pain, irritability, lethargy, vomiting, grossly bloody stools, diarrhea, altered consciousness, poor oral intake, abdominal mass, and duration of symptoms). The evaluation of patients and recording of data in the pediatric ER of our hospital were performed by pediatric residents, pediatric faculty members, emergency medicine residents, and emergency medicine faculty members. They used a standardized form, which consisted of historical and physical examination variables. Initial assessments were completed before any advanced diagnostic imaging [ultrasound (US), air enema fluoroscopy, or computed tomography (CT)] was conducted. When the clinical signs and symptoms signaled the presence of intussusception, an US was performed. The diagnosis of intussusception was confirmed when a target sign or doughnut sign was noted on the US; ileocolic intussusception was confirmed using air contrast enema. All diagnostic imaging findings were validated by a pediatric radiologist. The final diagnosis was based on the diagnosis of the hospitalized patient at discharge.

We defined fever as a temperature ≥38°C measured at home or in the ER. Paroxysmal pain was defined as severe, cyclic, and cramping abdominal pain, or episodic irritability. Non-specific abdominal pain was defined as non-severe and non-periodic abdominal pain that was not the typical paroxysmal pain. We defined bloody stool as grossly bloody spontaneously passed stool visualized by parents at home. Post-enema bloody stool was defined as the absence of stool until the ER visit with grossly bloody stool after glycerin enema in the ER. The classic triad included typical paroxysmal pain, bloody stool, and vomiting, but does not include post-enema bloody stool. The duration of symptoms was defined as the time from the onset of symptoms as determined by the parents to the ER visit. Door-to-diagnosis time was defined as the time from the ER visit to diagnosis by US/air contrast enema.

### Statistical Analysis

We performed an analysis by dividing all children into three categories according to age: <1 year, 1–2 years, and >2 years. The duration of symptoms was analyzed by dividing it into early and late groups, <6 h and ≥6 h, respectively. Based on initial assessment by the initial attending physician, the children were assigned to either the diagnosed or undiagnosed group. The undiagnosed group included patients who were discharged from the ER, admitted on the suspicion of other diseases, and patients who were diagnosed with intussusception based on the US or CT scanning after suspecting other diseases (for example, appendicitis) in the ER.

Continuous variables are presented as medians with ranges or means ± standard deviations. An independent Student's *t*-test or one-way analysis of variance was applied for continuous variables. Categorical variables were reported using frequencies and proportions and were compared using the Pearson χ^2^ or Fisher's exact test, as appropriate, or by linear association. The data were analyzed by using the Statistical Program for the Social Sciences (IBM SPSS Statistic Version 24, IBM Inc., Chicago, IL). A *P*-value of <0.05 was considered to be statistically significant.

## Results

A total of 536 patients were included in this study. The median age was 19 months (range, 2–132 months) and 345 (64.4%) patients were male. The classic triad of paroxysmal pain, vomiting, and bloody stool was encountered in 50/536 cases (9.3%). The median duration of symptoms was 11 h (range, 30 min−144 h) and the median door-to-diagnosis time was 2 h (range, 30 min-84 h).

### Comparisons by Age Group

Table 1 shows the clinical findings in each age group. Analyzing the three age groups, bloody stool, post-enema bloody stool, diarrhea, vomiting, poor oral intake, and lethargy were significantly more frequent in children <1 year (*P* < 0.05). In children ≥2 years, non-specific abdominal pain was significantly more frequent (*P* < 0.001), and the rate of undiagnosed intussusception was higher (*P* = 0.012). The duration of symptoms and door-to-diagnosis time were significantly longer in the group ≥2 years old than the groups <1 year and 1–2 years (*P* = 0.009 and *P* < 0.001, respectively).

**Table 1:** Clinical findings of enrolled patients in different age groups.

	**<1 year** ***n* = 174 (%)**	**1–2 years** ***n* = 187 (%)**	**≥2 years** ***n* = 175 (%)**	***P*-value**
Gender, M:F	112:62	112:75	121:54	0.351
Paroxysmal pain	120 (69.0)	164 (87.7)	131 (74.9)	0.191
Nonspecific abdominal pain	1 (0.6)	6 (3.2)	38 (21.7)	<0.001^a^
Vomiting	125 (71.8)	80 (42.8)	42 (24.0)	<0.001^a^
Bloody stool	78 (44.8)	34 (18.2)	12 (6.9)	<0.001^a^
**Post-enema (*****n*** **= 146)**				<0.001^a^
Bloody stool Normal stool	30 (78.9) 8 (21.1)	28 (46.7) 32 (53.3)	11 (22.9) 37 (77.1)	
**Classic triad of symptoms**				<0.001^a^
0 1 2 3	2 (1.1) 59 (33.9) 75 (43.1) 38 (21.8)	2 (1.1) 102 (54.5) 73 (39.0) 10 (5.3)	27 (15.4) 114 (65.1) 32 (18.3) 2 (1.1)	
Diarrhea	30 (17.2)	21 (11.2)	17 (9.7)	0.035^a^
Abdominal mass	13 (7.5)	10 (5.3)	13 (7.4)	0.989
Fever	21 (12.1)	30 (16.0)	29 (16.6)	0.239
Poor oral intake	62 (35.6)	45 (24.1)	27 (15.4)	<0.001^a^
Lethargy	45 (25.9)	16 (8.6)	16 (9.1)	<0.001^a^
Duration of symptoms (h)	15.8 ± 15.7	15.7 ± 17.2	21.1 ± 23.3	0.009^a^
Door-to-diagnosis time (h)	3.5 ± 5.2	2.4 ± 3.1	5.3 ± 10.3	<0.001^a^
Initial diagnosis (*n* = 428)				0.012^a^
Intussusception Other disease	132 (88.6) 17 (11.4)	134 (89.9) 15 (10.1)	101 (77.7) 29 (22.3)	

a *Significant difference by P < 0.05*.

### Comparisons by the Duration of Symptoms: Early vs. Late Group

**Figure 2:**
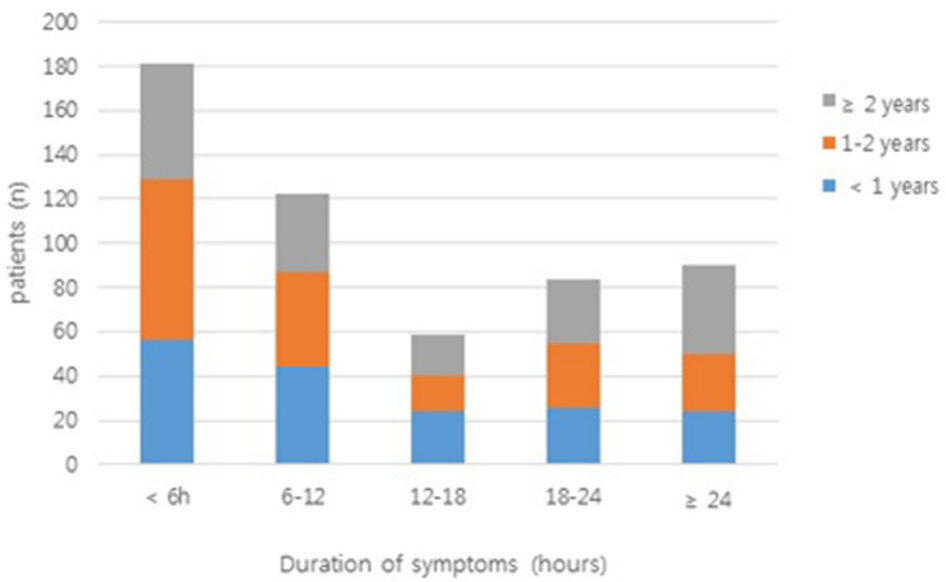
The duration of symptoms in patients enrolled in the study. Bars are colored according to the number of patients from each age group (<1 year, 1–2 years, and ≥2 years).

In a large proportion of patients (33.8%), the duration of symptoms was <6 h; in only 16.8% of patients, it exceeded 24 h (Figure 2). Table 2 shows the clinical findings of the early (<6 h) and late (≥6 h) groups. In the early group, paroxysmal pain was significant (*P* < 0.001). Bloody stool and fever were significantly more frequent in the late group than in the early group (*P* = 0.023 and *P* < 0.001, respectively).

**Table 2:** Clinical findings of enrolled patients in the early (<6 h) and late groups (≥6 h) classified based on the duration of symptoms.

	**<6 h** ***n* = 181 (%)**	**≥6 h** ***n* = 355 (%)**	***P*-value**
Gender, M:F	124:57	221:134	0.182
Paroxysmal pain	156 (86.2)	259 (73.0)	<0.001^a^
Nonspecific abdominal pain	14 (7.7)	31 (8.7)	0.745
Vomiting	77 (42.5)	170 (47.9)	0.272
Bloody stool	31 (17.1)	93 (26.2)	0.023^a^
**Post-enema (*****n*** **= 146)**			0.684
Bloody stool Normal stool	24 (40.7) 35 (59.3)	45 (51.1) 42 (47.7)	
**Classic triad of symptoms**			0.057
0 1 2 3	5 (2.8) 100 (55.2) 64 (35.4) 12 (6.6)	26 (7.3) 175 (49.3) 116 (32.7) 38 (10.7)	
Diarrhea	18 (9.9)	50 (14.1)	0.217
Abdominal mass	15 (8.3)	21 (5.9)	0.362
Fever	13 (7.2)	67 (18.9)	<0.001^a^
Poor oral intake	37 (20.4)	97 (27.3)	0.092
Lethargy	22 (12.2)	55 (15.5)	0.362
Duration of symptoms (h)	3.7 ± 1.6	24.6 ± 20.1	<0.001^a^
Door-to-diagnosis time (h)	3.6 ± 7.2	3.8 ± 6.9	0.73
**Initial diagnosis (*****n*** **= 428)**			0.667
Intussusception Other disease	132 (84.6) 24 (15.4)	235 (86.4) 37 (13.6)	

a *Significant difference by P < 0.05*.

### Comparisons by Diagnosis Status: Diagnosed vs. Undiagnosed Group

Of the patients included in this study, 108 were transferred from another hospital with a confirmed diagnosis of intussusception. A total of 428 patients were divided into diagnosed and undiagnosed groups according to the initial assessment and were compared. Of these, 367 were suspected of intussusception and 61 were not diagnosed with intussusception. In the diagnosed group, paroxysmal pain, bloody stool, post-enema bloody stool, and abdominal mass were significantly more frequent than in the undiagnosed group (*P* < 0.05) (Table 3). In the undiagnosed group, non-specific abdominal pain was significantly more frequent than in the diagnosed group (*P* < 0.001). There was no difference in the duration of symptoms in the two groups, but the door-to-diagnosis time was significantly longer in the undiagnosed than in the diagnosed group (*P* < 0.001). Table 4 shows the results of the initial assessment of the undiagnosed group. Twenty patients were hospitalized with other suspected diseases, and 41 returned to the ER with persistent or aggravated symptoms after discharge.

**Table 3:** Clinical findings of enrolled patients in the diagnosed and undiagnosed groups.

	**Diagnosed Group** ***n* = 367 (%)**	**Undiagnosed Group** ***n* = 61 (%)**	***P*-value**
Age (months)	19.6 ± 12.0	28.8 ± 20.1	0.001^a^
Paroxysmal pain	300 (81.7)	28 (45.9)	<0.001^a^
Nonspecific abdominal pain	15 (4.1)	18 (29.5)	<0.001^a^
Vomiting	177 (48.2)	31 (50.8)	0.782
Bloody stool	107 (29.2)	4 (6.6)	<0.001^a^
**Post-enema (*****n*** **= 146)**			<0.001^a^
Bloody stool Normal stool	61 (53.0) 54 (47.0)	3 (13.0) 20(87.0)	
**Classic triad of symptoms**			0.018^a^
0 1 2 3	10 (2.7) 172 (46.9) 144 (39.2) 41(11.2)	12 (19.7) 35 (57.4) 13 (21.3) 1(1.6)	
Diarrhea	51 (13.9)	7 (11.5)	0.691
Abdominal mass	29 (7.9)	0 (0.0)	0.023^a^
Fever	48 (13.1)	8 (13.1)	1.000
Poor oral intake	96 (26.2)	14 (23.0)	0.639
Lethargy	58 (15.8)	8 (13.1)	0.704
Duration of symptoms (h)	16.0 ± 17.7	18.0 ± 20.8	0.436
Door-to-diagnosis time (h)	2.3 ± 2.0	16.3 ± 15.1	<0.001^a^

a *Significant difference by P < 0.05*.

**Table 4:** Initial assessment of patients in the undiagnosed group.

	***N* = 61**
Acute gastroenteritis	35
Fecal impaction	19
Appendicitis	4
Meningitis^a^	2
Henoch-Schonlein purpura	1

a *Two children (6 and 12 months old) showed only mental changes but no gastrointestinal symptoms*.

## Discussion

Intussusception is notoriously difficult to diagnose because of the lack of sensitive or specific clinical indicators. However, it is a potentially dangerous disease that can lead to death if left untreated, and it is therefore imperative to prevent underdiagnoses of patients with suspected intussusceptions. This study showed differences in clinical features of ileocolic intussusception with the duration of symptoms and age of the children. In addition, this study analyzed the diagnosed and undiagnosed patients, as determined by the initial attending physician. We also identified cases in which it was difficult to diagnose intussusception and noted various symptoms that are important in preventing underdiagnoses.

In our study, the most common symptom of intussusception was paroxysmal pain, which was observed in 77.4% of patients, regardless of age. Previous studies have reported abdominal pain in 13–22% of patients (13, 14). Our results showed a higher percentage compared to previous studies because our definition of paroxysmal pain included both typical abdominal pain and episodic irritability. In younger children, especially those under 1 year, typical abdominal pain may be mistaken for episodic irritability because they are unable to express themselves verbally. Typical paroxysmal pain is described as severe, cyclic, cramping, abdominal pain, or irritability. Episodes may persist for 4 to 5 min interspersing with a relief period of 10 to 20 min. This paroxysmal pain is an important early indicator that suggests parents to take their child to the ER; therefore, paroxysmal pain is significantly higher in the early (<6 h) than in the late group (≥6 h) in the current study (*P* < 0.001).

The second most common symptom was vomiting (46.1%, *n* = 247). Vomiting was significantly more common in younger children (*P* < 0.001), similar to previous studies (3, 15). Vomiting is caused by a vagal reflex and ileus condition. Older children may have better control of the vomiting reflex, which could be why vomiting was less common in older children.

Bloody stool, though a key feature of intussusception, was less common than other symptoms. Its frequency varied considerably between studies, with reported rates ranging from 9 to 36% (16–18). In our study, only 23.1% (*n* = 124) of all patients had bloody stool, and it was more common in younger children (44.8, 18.2, and 6.9% in the <1, 1–2, and ≥2 year age groups, respectively, *P* < 0.001). This result was comparable with that of previous studies (19, 20). Yamatomo et al. reported that spontaneously passed grossly bloody stool was more common in the younger age groups (72, 53, 18, and 18% in the <8 month, 8–15 month, 16–35 month, and >3 year age groups, respectively, *P* = 0.0001) (20). Newman et al. reported a bloody stool rate of 96% in their studies in children younger than 4 months with intussusception (21). In our study, post-enema bloody stool was also more common in younger children (*P* < 0.001), and children older than 2 years tended to have a normal stool (no blood in the stool) even after glycerin enema (77.1%). Bloody stool may occur as a result of the compression of the mesenteric vessels, mucosal edema, hemorrhage, and ischemia of the bowel, and may not be detected in early cases (15, 19). Bloody stool was significantly more common in the late than in the early group (*P* = 0.023). This indicates that bloody stool is highly specific for intussusception, but an absence of bloody stool cannot exclude the probability of the presence of intussusception.

The triad of symptoms, including paroxysmal pain, vomiting, and bloody stool, is described in textbooks as the classic triad of intussusception, but several previous studies have reported these symptoms in only 10 to 20% of cases (2, 16, 19). In the current study, 9.3% (50/536) of children showed the classic triad of symptoms, and only two (1.1%) of them were over 2 years. Harrington et al. reported that the combination of the classic triad symptoms showed a positive predictive value of 93% (*P* < 0.001) (22), but in many children, especially in early cases and older children, the classic triad cannot be the sole diagnostic criteria for intussusception (3, 15, 16).

In this study, poor oral intake and lethargy were significantly higher in younger children than in older children. Vomiting and decreased oral intake can lead to severe dehydration and shock, as well as neurological symptoms such as altered mentality, lethargy, and seizures that may appear as the initial presentation. In our study, two children were misdiagnosed with meningitis due to mental changes without gastrointestinal symptoms and were later diagnosed with intussusception. Dominguez-Carral et al. reported that fewer than 3% of children with intussusception initially presented with isolated neurological symptomatology (lethargy being the most common symptom) out of 351 cases (23). Maldonado et al. recommend checking the stool for occult blood and performing an abdominal US in undiagnosed patients with altered mental status, particularly in those younger than 12 months (12).

In the current study, children older than 2 years had a longer duration of symptoms and door-to-diagnosis time than the other two groups, and were more likely to be undiagnosed. Turner et al. and Kim et al. also reported that children older than 2 years were prone to receiving a delayed diagnosis (24, 25). Older children were more likely to have non-specific abdominal pain than in younger children, and the diagnosis was often difficult. Whereas, bloody stool or vomiting were relatively common in younger children, resulting in parents visiting the ER early, with intussusception quickly being suspected.

When comparing the diagnosed group with the undiagnosed group, the decision to perform an additional image study was easy when there was paroxysmal pain, bloody stool, and abdominal mass, indicative of intussusception. Conversely, in the case of non-specific abdominal pain, other diseases were considered first. When 146 children who did not spontaneously pass bloody stool underwent glycerin enema in the ER, 64 in the undiagnosed group and more in the diagnosed group had bloody stool (*P* < 0.001). This finding suggests that glycerin enema also helps diagnose intussusception in children without the typical triad of symptoms. A negative post-enema bloody stool cannot exclude intussusception but shows that it as a significant positive predictor. Door-to-diagnosis time in the undiagnosed group was significantly longer than that in the diagnosed group (*P* < 0.001), showing how important it is to suspect intussusception for timely diagnosis treatment.

For early detection of intussusception, a few recent studies have suggested that bedside US by radiologists and emergency physicians can be an effective diagnostic modality (26, 27). However, it is not routinely available in all clinical settings and is highly operator-dependent. Several studies have included the findings of abdominal X-rays as diagnostic criteria for intussusception (7, 28). However, their diagnostic value remains unclear, with a sensitivity of only 29–50% and the disadvantage of radiation exposure. Definitions and criteria for radiological findings differ for each institution. In our ER, the interpretation of abdominal X-rays by pediatric radiologists was not always available, and attending physicians were forced to discern the possibility of intussusception based on clinical symptoms rather than abdominal X-rays. Therefore, we did not include abdominal X-rays in the analysis.

This study had some limitations. First, this was a retrospective study, and the quality of data collected was dependent on medical records. A standardized form consisting of historical and physical examination variables was used, but data might not be complete or accurate. Second, we divided the diagnosed and undiagnosed groups, based on the clinical suspicion of the attending physician in the ER. Clinical suspicion and assessment can be subjective, as there are multiple attending physicians and clinical experience varies from person to person. This may have affected the analysis of the diagnosed and undiagnosed groups. Finally, this study had no control group, so our data are presented only as absolute numbers and percentages. We cannot comment on the sensitivity, specificity, or positive and negative predictive values of our findings.

In conclusion, the clinical findings of ileocolic intussusception may vary depending on the duration of symptoms and age of the children. If children, especially those under 1 year, have paroxysmal pain, vomiting, bloody stool, poor oral intake, or lethargy, intussusception should be suspected. It is important to keep in mind that in older children, non-specific abdominal pain without bloody stool may be a symptom of intussusception. Glycerin enema also helps in diagnosing intussusception in children with no typical symptoms. Diagnosis of intussusception is still difficult, but carefully identifying and considering all symptoms is essential for timely diagnostic treatment in children. We hope that this study will aid in the timely and appropriate treatment of children of various ages with varying symptoms.

## Data Availability Statement

The datasets used and/or analyzed during the current study are available from the corresponding author on reasonable request. Requests to access these datasets should be directed to mjtolord@hanmail.net.

## Ethics Statement

The studies involving human records were reviewed and approved by Ulsan University Hospital Human Research Ethics Committee.

## Author Contributions

MC: study conception and design, critical revision, drafting of the manuscript. IP and MC: analysis, data interpretation, and data acquisition. All authors: contributed to the article and approved the submitted version.

## Conflict of Interest

The authors declare that the research was conducted in the absence of any commercial or financial relationships that could be construed as a potential conflict of interest.

## Abbreviations

ER: emergency room
US: ultrasound
CT: computed tomography.
